# Design Considerations for a 120 GHz MIMO Sparse Radar Array Based on SISO Integrated Circuits

**DOI:** 10.3390/s25185622

**Published:** 2025-09-09

**Authors:** Luigi Ferro, Changzhi Li, Emanuele Cardillo

**Affiliations:** 1 Department of Engineering, University of Messina, 98166 Messina, Italy; 2 Department of Electrical and Computer Engineering, Texas Tech University, Lubbock, TX 79409, USA; changzhi.li@ttu.edu

**Keywords:** antenna distance, chips positioning, D-band application, millimeter-waves, MIMO radar, sparse array

## Abstract

This study aims to illustrate the main aspects of designing a modular 120 GHz Multiple-Input Multiple-Output (MIMO) sparse radar array (SRA) composed of multiple Single-Input Single-Output (SISO) Integrated Circuits (ICs). Although the scientific literature reports on 120 GHz integrated circuit prototypes, to the authors’ best knowledge, there are no commercial MIMO radars composed of multiple SISO ICs operating in the D-band spectrum. The design involves many challenges; indeed, the necessity to combine multiple chips with fixed dimensions and the presence of transmitting and receiving antennas on chips add many constraints for the antenna placement and, consequently, for the virtual array design. As an example, the minimum distance between the antennas must be at least equal to the chip width, which is in turn higher than half a wavelength and renders the array into a sparse configuration, thus raising many concerns regarding fixing the optimum inter-chip distance. Thus, this contribution can be considered as pioneering, being focused on the emerging concept of designing D-band MIMO radars by exploiting a modular approach.

## 1. Introduction

Nowadays, MIMO radars represent a key technology to sense the monitored environment due to the ability to estimate the angle of the target in respect to the radar position [1,2,3]. Generally, the evaluation of the target distance and its velocity is considered a well-consolidated task within the scientific community [4,5], but radar systems are also used for their ability to estimate speed and millimetric displacements relative to the targets of interest for many applications [6,7,8,9]. For instance, several researchers have focused their efforts on developing touch-free systems or exploring the detection of the targets based exclusively on micro-Doppler signature [10,11,12,13,14].

The angle estimation capability paves the way to an in-depth and accurate analysis of the surrounding area, both on the azimuthal plane and the elevation one. This ability is based on the presence of multiple antennas both in the transmitting and receiving channels. Moreover, MIMO radars provide information on the target in a single snapshot, thus overcoming typical cons like waiting time and processing effort to carry out a complete scan in the case of synthetic aperture radar (SAR) [15,16,17,18]. Of course, the use of MIMO radar is restricted to analyzing relatively limited areas in short-range applications, whereas SAR is employed to create large-scale maps of regions on Earth for several different applications [19,20,21].

In addition, the research is focused on developing radar in the license-free Industrial Scientific Medical (ISM) band centered around 122.5 GHz. The higher frequencies allow the miniaturization of antennas, which can be realized on-chip or in-package and, thus, enabling the implementation of highly integrated and cost-effective MIMO radar systems too. At the same time, the larger the bandwidth, the better the range resolution.

All these aspects are continuously increasing the interest of the scientific community in the MIMO radar field. In [22], the researchers put efforts into designing an entire monolithic microwave integrated circuit (MMIC) chipset oriented to MIMO radar application; instead, in [23], the authors worked hard to design a multichannel D-band receiver for MIMO radar with an optimized local oscillator (LO) distribution.

When designing a MIMO radar, one of the most critical sections is the antenna design, especially at higher frequencies. The authors of [24] presented the design of a planar antenna array through a method based on the definition of element array position by a genetic algorithm and the analysis of the ambiguity function associated with the array itself. This work was carried out for a radar system with a center frequency of 76.5 GHz, enabling both azimuth and elevation environment monitoring.

Once again, the scientific interest is also confirmed by several research groups who focused on realizing new microstrip antennas tailored for millimeter-wave automotive MIMO radar sensors, as in [25]. Another example is present in [26], where radio frequency (RF) front-end was realized in a modular way, considering 24 transmitters and 24 receivers.

Our idea is to create a MIMO sparse radar array (SRA) exploiting multiple commercial transceivers already available on the market. This contribution wants to formalize the considerations and steps necessary to make a modular millimeter wave MIMO radar array. To the best of the authors’ knowledge, this is the first project of a modular MIMO radar array operating at 120 GHz and composed of multiple cooperating integrated circuits. Indeed, most commercial MIMO radars work within the industrial and automotive bandwidths around 60 and 77 GHz, respectively.

As already mentioned, the fundamental component of the MIMO radar is the number of antennas and their arrangement. In addition, the idea is to exploit higher frequencies because of the aforementioned advantages. In the D-band spectrum, the transceiver antennas are usually on-chip; this is a beneficial feature to reduce the dimension, but it raises additional concerns because the antenna position is strictly related to the chip position and the antennas are inside the package itself. Typically, researchers design new array topologies by optimizing the position of each element to improve the array performance. Different algorithms can be used [27,28], and the research community is continuously developing new algorithms, either to optimize specific aspects of arrays or to exploit sidelobes and grating lobes in novel ways [29,30]. However, this contribution applies a different approach to elude the limitations due to the physical dimensions of the chips and to optimize any performance parameters in the MIMO radar field.

As explained in the next paragraph, one of the main performance parameters in the case of MIMO radar, such as angular resolution and field-of-view (FOV), is mainly dependent on the wavelength, the number of array elements, and the distance among them. Developing a D-band MIMO radar array becomes an extremely challenging task because the wavelength is smaller than the chip dimensions. The placement of the various antennas at the classic distance of λ/2 is impossible because the chip width itself is larger than λ/2. For this reason, it is mandatory to outdistance the antennas by moving away the chips and overcoming the λ/2 limit. This causes the well-known growing presence of nulls in the radiation pattern [31] and, in MIMO radar applications, degraded performance. The next section analyzes these issues to find a trade-off that would enable the realization of a D-band MIMO radar.

To summarize, the novelty of the contribution relies on the design of the first modular D-band MIMO sparse radar array. A crucial aspect is to investigate how MIMO radar performance changes in relation to the presence of on-chip antennas and how the restriction due to the antenna position can be exploited to enhance the radar performance. Last but not least, the willingness to use multiple SISO radar ICs will drastically decrease the design and future realization costs.

The paper is organized as follows. The theory and key performance parameters of MIMO radar have been discussed in the first part of Section 2. The second part of Section 2 shows how the performance changes depending on the antenna arrangement and how to exploit different configurations. The theoretical analysis has been confirmed by means of simulations in Section 3. Finally, the conclusions are drawn in Section 4.

## 2. Performance Criteria and Array Arrangement

### 2.1. MIMO Radar Array Theory

**Figure 1:**
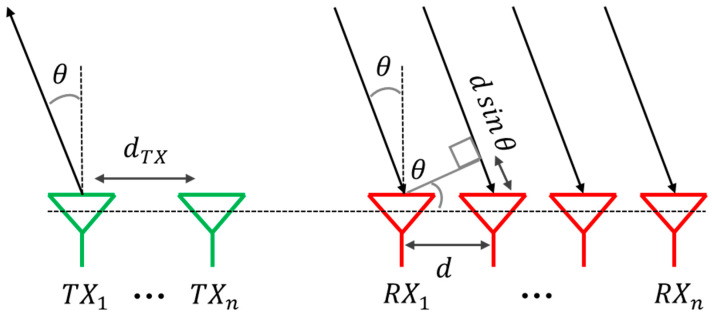
Angle estimation with a generic MIMO radar.

The main goal of a MIMO radar array is to detect the Angle of Arrival (AoA), i.e., to estimate the target’s precise location across the environment near to radar itself. In other words, the presence of transmitting and receiving antennas at a defined distance is the main requirement to evaluate the spatial position of the target, as highlighted in Figure 1.

Considering θ as the angle that must be estimated, at least one transmitting antenna and two receiving antennas are necessary. Indeed, the task is to calculate the extra distance, dsinθ, that the received echo related to a subsequent antenna travels compared to the echo received by the previous antenna. Thus, the AoA can be calculated as in the following Equation (1):

(1) $$\theta =\sin^{-1}\left(\frac{\varphi \cdot \lambda }{2\pi \cdot d}\right)$$

where φ is the phase shift between two received echoes of consecutive received antennas due to the extra distance. Of course, λ is the wavelength and d is the distance between elements in the receiving section. Two important merit criteria in the MIMO field are the FOV and the angular resolution. It is worth noting that the unambiguous FOV is defined as the angular volume in which the radar is able to detect a target. Angular resolution is defined as the minimum detectable angular difference between two different targets at the same range and speed. The π-limit, to have an unambiguous measurement, affects the FOV of the MIMO radar system and can be expressed as in (2) by replacing φ with π.

(2) $$FOV={\pm \sin}^{-1}\left(\frac{\lambda }{2\cdot d}\right)$$

**Figure 2:**
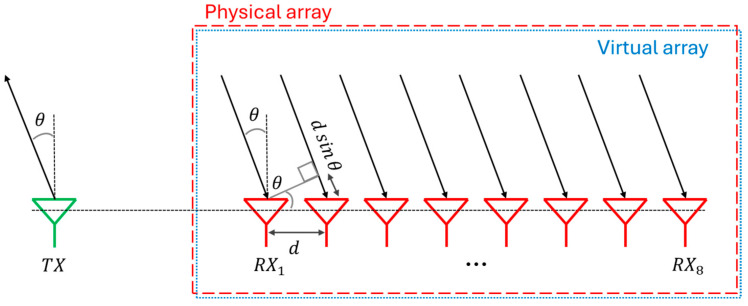
Example of a physical array.

Normally, the inter-antenna distance is restricted to λ/2 to limit the presence of grating lobes in the radiation pattern. It follows, generally, that the FOV ranges from −90° to +90°, whereas the angular resolution can be defined in degrees as:

(3) $$\theta_{res}=\frac{180{}^{\circ}}{\pi }\;\frac{\lambda }{N_{array}\cdot d\cdot \cos\theta }$$

where $N_{array}$ is the number of elements in the array. This expression can be simplified by making two assumptions: the inter-antennas distance equal to λ/2 and cosθ=1 in case of a boresight view, i.e., θ=0. In this way, the angular resolution depends exclusively on the number of elements. A good resolution is necessary to discriminate between more targets within the scenario. These equations are only valid for uniform array; otherwise, different considerations should be drawn. In order to deeply understand how to improve these performance parameters, the concept of virtual array must be introduced. In summary, each transmitting signal from each antenna in transmission will be captured by every antenna in the receiving section. As can be trivially noticed in (3), the greater the number of receiving elements, the better the resolution. At first glance, increasing the number of antennas in the receive section could be a good choice, as highlighted in Figure 2. Unfortunately, this operation has a huge impact on the size, cost, and complexity of the receiver due to the increasing number of low-noise amplifiers (LNAs), mixers, filters, and analog-to-digital converters (ADCs) for each receiving channel.

**Figure 3:**
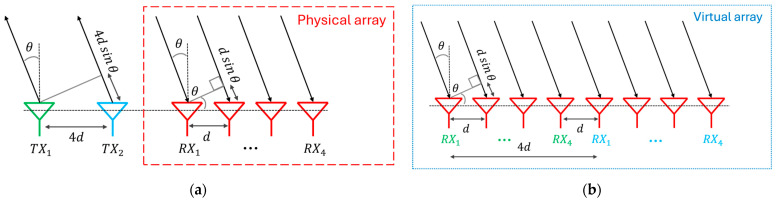
Physical array (**a**) and corresponding virtual array (**b**).

Consequently, the best solution is to increase the number of transmitting antennas, as illustrated in Figure 3.

Therefore, the physical array in Figure 3a consists of two transmitting and four receiving antennas. The transmission from the first antenna results in four received signals, each of which is out of phase with the previous one by a quantity of φ. The second transmission antenna is placed at 4d from the first one; thus, any signal from *TX*_2_ travels an additional path of length 4dsinθ respect to the path due to *TX*_1_. The signal phase of the first receiving antenna due to the second transmission is 4φ, but only φ more than the phase of the last echo generated from the first transmission because of the d distance between the last antenna of the first transmission and the first antenna of the second one. Thus, Figure 3b shows the virtual array that has been created with the identical structure and characteristics of the physical array in Figure 2. The number of physical receiving antennas is four, but the addition of a second transmitting antenna allows us to simulate the presence of four different receiving antennas if they are placed properly in the array. In this way, the angle resolution capability can be improved by adding virtual antennas while keeping relatively low the hardware complexity. In fact, the system in Figure 2 presents nine path channels (one transmitting and eight receiving), three more than the other introduced system.

If the first array were composed of only four receiving antennas instead of eight, the angle resolution of the virtual array would have been better than the physical one. Trivially, the aperture of the virtual array would have been larger than the physical size of the real array.

This affirmation suggests that angular resolution depends on array size rather than the number of elements in the array. This is confirmed by the fact that an increasing number of array elements leads to an extension of the array size. Therefore, according to [32], the Rayleigh criterion can be used to determine the virtual array length and, thus, the angular resolution as in the following Equation (4):

(4) $$\theta_{Rayleigh-res}\approx \frac{180{}^{\circ}}{\pi }\;1.22\;\frac{\lambda }{d_{virtual}}$$

where $d_{virtual}$ is the array total aperture that can be calculated by summing the distances among each element of the virtual array or the addition of the position of the last physical transmitter plus the last physical receiver’s one minus the position of the first physical receiver.

This expression is valid for both the uniform array and the non-uniform one. Generally, a larger antenna aperture must be designed to achieve a better angular resolution. For instance, this can be achieved by using element spacings of λ instead of the conventional λ/2 at the cost of a reduced FOV.

In summary, virtual antennas behave like additional physical antennas. This keeps hardware complexity to a minimum, thus providing a cost-effective way to enhance the angle resolution capabilities. The same virtual array can be realized by combining different distances between the *TX* and *RX* antennas. Therefore, the final choice may depend on how easily the array can be arranged on the radar board.

Based on the listed parameters, the next paragraph is focused on two main integrated circuit configurations to determine their best position to optimize the performance.

### 2.2. Chips Arrangement

The chip selected for this project is the TRA_120_45 transceiver [33] by Indie Semiconductor, formerly Silicon Radar, 5 × 5 mm^2^ in size and characterized by the presence of one transmitting and one receiving dipole antenna on-chip. The operative frequency can be chosen in the range of 112 GHz–137 GHz. The best choice in this context is to use a lower frequency, which results in a higher wavelength value. For the sake of simplicity, the relatively low central frequency of 120 GHz has been chosen to make calculations with a 2.5 mm wavelength, exactly half the chip width, i.e., 5 mm. The presence of on-chip antennas eliminates the need for bond wires at this very high operating frequency. The bond wires are practical for frequencies superior to 100 GHz, but the optimum length of the bond wire does not correspond to the optimum radiation pattern as presented in [34].

Therefore, the size of the transceiver has a significant impact on the positioning of the antennas because they are within the chip itself; indeed, FOV and angular resolution are affected by their spatial position. The ideal case is to have the maximum possible FOV and the minimum angular resolution in order to detect more targets in a very wide angular range. Unfortunately, this is not a trivial achievement since the higher the distance among antennas, the better the angular resolution, but the worse the field-of-view. Thus, the main critical point is the size of every chip because the minimum distance between the antennas, d, must be at least equal to the chip width, i.e., to 2λ in case of a 5 × 5 mm^2^ integrated circuit. So, the main constraint is the minimum antenna distance due to chip dimensions. Indeed, the antenna position is fixed inside the transceiver. This physical and more stringent limit does not permit the application of either of the two methods proposed in [35], where a two-step synthesis procedure is used to determine antenna array sparsity via convex optimization. The physical limitation makes it difficult to use the stochastic search method to find a very good nonuniform distribution for the array elements as presented in [36], where the goal is to find the distribution and weighting values of the array elements to minimize the sidelobe level.

Generally, the angular resolution can be improved by increasing the number of elements in the array, as seen in (3), while keeping the λ/2 element gap unchanged. In this contribution, the idea is to exploit the 2λ constraint to achieve a superior angular resolution; however, at the cost of a reduced FOV. In Table 1 the variation in merit criteria has been reported at varying distances.

**Figure 4:**
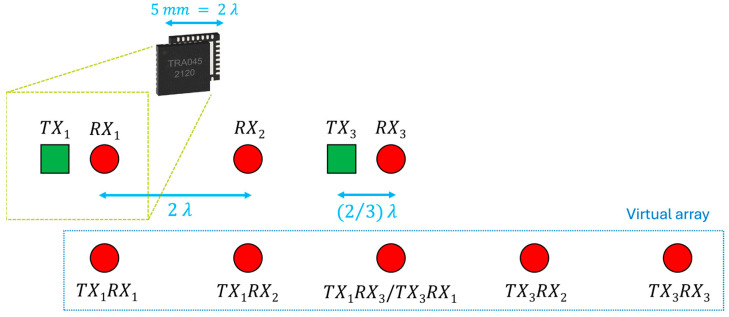
Chips placement and its virtual array. The green squares are the transmitting antennas, the red circles identify the receiving ones. On the top left the tiny representation of the selected chip.

Table 1 highlights clearly that the field-of-view drastically decreases by increasing distance; instead, the resolution improves, but not as clearly. The resolution at 2λ distance is already very good. Therefore, the idea is to maximize target recognition by spacing three integrated circuits of 2λ apart. The choice of three ICs was dictated by the willingness to lower the project complexity and to develop a MIMO radar operating along the azimuth plane at this first stage exclusively. A preliminary configuration is shown in Figure 4 in which the antennas are illustrated nearly as point-like in a uniform arrangement.

The green square shapes represent transmitting antennas, and the red circles identify the receiving ones. Every shape couple can be assimilated with the antennas inside each chip, which is represented with a gold hatching in Figure 4. The distance between the transmitting antenna and the receiving one within a single transceiver is (2/3)λ; that is, this value is the distance between the λ/4 elements of the half-wave dipole. In this configuration, there are only two transmitting antennas to avoid overlapping elements in the virtual array. If the second transmitter were used to radiate the signal, the generated virtual elements would be useless because they would present element duplication. Moreover, an energy part is saved by disabling one transmitter. Therefore, the position of each virtual element remains unchanged; the number of overlapped elements has been reduced solely. It is worth pointing out that it will be necessary to increase the distance d a little bit more than 2λ to avoid rubbing the chips. Nevertheless, d is considered equal to double the wavelength to simplify calculations and analysis. Therefore, it results a 2λ distance among virtual elements starting from the first element at position 0 to the last one at position 8λ. The 2λ corresponding FOV and $\theta_{res}$ are present in Table 1, whereas the angle resolution is 8.74° by applying (4). The angular resolution checking will be performed in the next section by simulating the array pattern.

**Figure 5:**
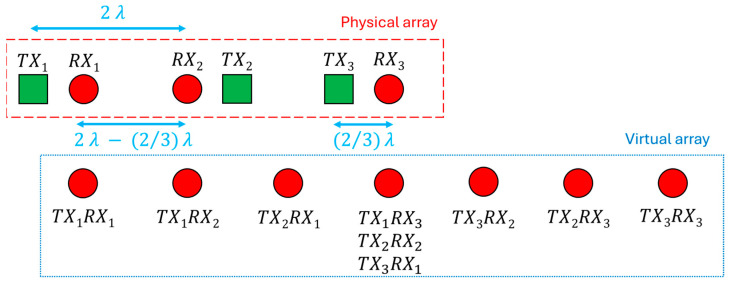
Chips placement and its virtual array in case of the second chip rotation. The green squares are the transmitting antennas, the red circles identify the receiving ones.

Another possibility is to investigate a chip arrangement characterized by the alternating 180° rotation of the chips themselves, as shown in Figure 5.

The alternating chip 180° rotation changes the inter-antenna distance between odd and even chips but maintains constant the distance between the elements of the virtual array, i.e., the arrangement seems non-uniform only apparently. The position of every virtual element with respect to the first one is reported hereafter: 0, 2λ−2/3λ, 2λ+2/3λ, 4λ, 6λ−2/3λ, 6λ+2/3λ, 8λ. Thus, the virtual inter-antenna distance is equal to (4/3)λ and the array is still uniform. It is curious to underline that the corresponding virtual array of each transmitting antenna is non-uniform, but all their combinations generate an equally spaced array. In other words, it seems as if each singular non-uniform array would cover the non-uniformity of the others, making uniform the entire virtual array.

It is immediately noticeable that the number of overlapped antennas is less than the previous arrangement if the second transmitter is not disabled. This is due to the different spacing between the transmitting and receiving antennas. Therefore, in this configuration the second transmitter is used because it adds active elements that improve the array performance significantly. For this reason, in the first configuration it is useful to disable one transmitter to save energy at least, while activating each transmitter to improve performance in the second configuration.

The rotation of the chip changes only the internal arrangement of the elements, but not the total aperture size, which remains equal to 8λ, as in the previous case. According to (4), it is expected to have an identical angular resolution. The resolution is 6.14° by calculating with (3). Notwithstanding the minor virtual elements distance, the resolution is very similar to that of the previous antenna arrangement, thanks to the increasing number of element in the virtual array, which partially counteracts the resolution deterioration. According to (1), the FOV is ±22.02° which is 50% higher than the first array arrangement due to closer antennas.

Therefore, the negligible worsening of angular resolution is acceptable because it implies a significant improvement in field of view, even though it is still relatively small.

Therefore, a list of main points is reported below:

- minimum distance among chips equal to 2λ in case of TRA_120_45 transceiver;
- the higher the inter-chip distance, the worse the FOV, but the better the angular resolution;
- there is very little difference in terms of resolution between the two different array topologies;
- a 50% FOV improvement by rotating the second integrated circuit.

## 3. Simulation and Final Considerations

A simulation of the array factor is crucial to confirm the calculated angular resolution and estimate the angular position of the grating lobes. Indeed, the angular resolution can also be defined as the 3 dB beamwidth of the array main lobe. It is also correlated with the ratio between wavelength and array dimensions [37].

The element spacing among the antennas directly impacts the presence and location of grating lobes [31,38]. The expected location of the first grating lobe can be calculated by means of (5).

(5) $$\theta_{GL}\approx \sin^{-1}\left(\frac{\lambda }{d}\right)$$

Thus, the first arrangement presented in this contribution will have the top grating lobe at approximately 30°, whereas the 180° rotation solution will be characterized by a first grating lobe in 48.59° angular location.

**Figure 6:**
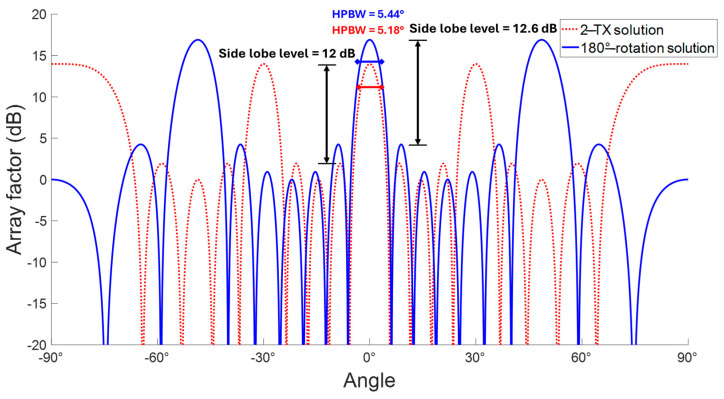
Main lobe width, grating lobes, and side lobe level of both uniform arrays.

The results are corroborated by means of the simulation, and the array factor comparison is shown in Figure 6.

The red dotted line represents the array factor generated by the two activated transmitters and three receivers illustrated in Figure 4, whereas the blue solid line is related to the solution obtained after the 180° rotation, where the second chip is rotated with respect to the other two as highlighted in Figure 5. Regarding the side lobe level, the second array exhibits a slightly lower level, especially near the main lobe. In addition, the blue array factor has a higher gain due to its superior number of virtual elements. Specifically, the red array presents a gain factor equal to 14 dB, instead of 17 dB for the blue array. Thus, even though the sidelobes of the blue array are slightly superior to the red ones, the total sidelobe level is favorable in the case of the blue array by a small margin. It is also characterized by fewer grating lobes that are farther away from the main lobe than the red one. This last aspect is corroborated by the fact that the larger the inter-antenna space, the higher the number of grating lobes; indeed, the inter-distance is (4/3)λ for the blue array and 2λ for the red one. In addition, the simulation confirms the angular location of the first grating lobe for both array factors, as mentioned just above. Hence, the blue sidelobe section is larger than the red one, which ensures that the grating lobes are more distant with respect to the main lobe in the blue array factor. Furthermore, the red array factor does not have a grating lobe at an angular location of 90°. Instead, the blue array factor does not have another pronounced grating lobe after the first one. Of course, the same considerations can be drawn for the specular sections of both array factors.

The angular resolution can be extracted by simulation considering the half-power beamwidth (HPBW) as highlighted in Figure 6. The red beamwidth is 5.18° and the blue one is 5.44°. These widths validate the ones calculated with (3), 5.73° and 6.14°, respectively. At first glance, the (4) does not fit very well with the simulated results. The simulation reinforces the idea that the larger inter-distance of the first solution ensures marginally better angular resolution. Indeed, the higher element number of the second solution covers, partially, the resolution worsening due to the minor inter-antenna span. Therefore, the angular resolution cannot be considered as a discriminating factor to make a choice between the two factors.

In summary, the best solution involves the second type of arrangement with respect to the first one for the following reasons:

- lower side lobe level;
- less cumbersome presence of grating lobes;
- angular resolution comparable to the other presented solution;
- significant increase in terms of FOV.

**Figure 7:**
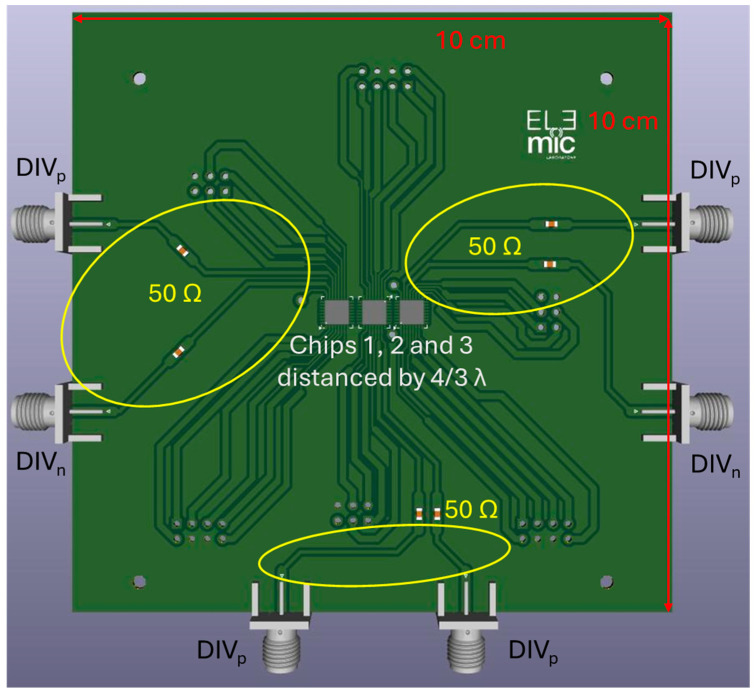
First prototype layout of the MIMO radar system.

It can be asserted that the 180° rotation is a smart solution to widen FOV without complicating the project. The next step was designing the board layout. The RO3010 substrate was chosen [39], and the layout was drawn in KiCad, which is the electronic design automation software used for this project. The SISO ICs were placed based on all the considerations made in the previous chapter. Above all, attention was given to the microwave section and the placement of headers and coaxial connectors to ensure mechanical stability. The design layout is sketched in Figure 7. Specifically, the board has a length and a width of 10 cm to incorporate the chips and headers, which are not illustrated in their 3D models to represent only the key information in Figure 7. There are six microwave paths, two for every chip on the board, to make the divider outputs available for each transceiver. These outputs can be exploited to check that every transceiver is working properly when the radar board is realized, for example, using a spectrum analyzer. It is expected that the divider output frequency ranges from 1.73 GHz to 2.15 GHz, as confirmed in [33]. A capacitor is present in every microwave section because an external decoupling capacitor is required, as can be noted in the datasheet. Consequently, these brief microstrip paths were designed by using the AWR Design Environment Platform by Cadence, ensuring a 50 Ω impedance as much as possible along each microwave path to obtain an input reflection coefficient below the value of −10 dB [40]. Additionally, the microwave connectors were arranged with that orientation to guarantee mechanical stability of the board. The chips were placed in the center to have more space around them to facilitate the path of each signal. For the sake of brevity, the simulation of the microwave section has not been reported. After the realization of the board, some measurements will be taken to confirm that everything works correctly.

Despite being a different contribution, this work and [24] try to design an array for MIMO applications with a different approach and tasks. It is worth noting that this contribution aims at designing a 120 GHz MIMO radar, which is more challenging compared to the 76.5 GHz operative frequency in [24] due to the small wavelength that complicates the array design. Moreover, the estimated angular resolution in the azimuth plane is 15.15°, quite higher than 6.14° of this project. Our solution is characterized by a lower side lobe level, around 6 dB vs. 12 dB in absolute value. In any case, in [24], the researchers applied a genetic algorithm that is specialized for a 2D MIMO radar optimization, in which a gene represents the antenna element position on the plane. All the genes represent a single full 2D MIMO array configuration, the so-called chromosome. Consequently, they set some constraints in the algorithm to optimize the performance. Once again, the comparison cannot be deepened further because this contribution focuses on exploiting the physical dimensions of each chip as an advantage to cut design costs rather than designing an array using a new algorithm.

Similar considerations can be drawn comparing this contribution to [36,38]. In [36], the nonuniform distribution of the array elements was optimized by means of a stochastic search method. The goal was to find the distribution and weighting values of the array to obtain the minimal sidelobe level with some constraints for the main lobe location and width. In [38], a similar MIMO radar was designed. Even here, the researchers positioned the array elements freely, without any physical constraints. Consequently, their work is different from this contribution, which addresses the possibility of using already existing transceivers on the market.

In any case, continuous research is conducted to find a balance among several parameters, limitations due to the application, and those imposed by the recommended solution. This comparison highlights that the results of this contribution are comparable to the previous research [38] merely in spite of very strict physical limitations and by employing commercial transceivers. Thus, no additional RF design efforts were necessary.

Finally, the large available bandwidth of the transceiver could be exploited to suppress aliases caused by grating lobes, i.e., the use of different sub-bands can allow us to distinguish targets due to the main lobe with respect to the ones generated by grating lobes [41], mitigating their effect on this project too.

## 4. Conclusions

This article outlines the main steps required to realize a MIMO sparse radar array composed of ready-to-use SISO integrated circuits. This work did not follow an existing algorithm for designing the array due to the physical dimensions of the chip and the position of the antennas within the package itself. This different approach permits cutting the costs of designing a MIMO array that operates in the D-band spectrum. Hence, the main critical aspects have been analyzed to recommend a concrete solution. Of course, several other aspects must be considered in the next phases of the design, like chip synchronization, the design of a suitable baseband board, and the acquisition and management of the data samples via a microcontroller or FPGA. In addition, it will be essential to apply a calibration technique to the sparse array in order to compensate for the amplitude and phase offsets among the signals received by each antenna. This contribution can be exploited by other researchers to focus on the main aspects required to design a modular millimeter wave MIMO radar.

## Figures and Tables

**Table 1:** Effect of the distance variation on field-of-view and angular resolution.

d	FOV	$\theta_{res}$
2λ	±14.48°	5.73°
3λ	±9.59°	3.82°
4λ	±7.18°	2.86°
5λ	±5.74°	2.29°

## Data Availability

The original contributions presented in this study are included in the article. Further inquiries can be directed to the corresponding author(s).
